# Foreign

**Published:** 1841-08-14

**Authors:** 


### FOREIGN.

*On Electrical Currents in the Nerves.* By Professor Bischoff of Heidelberg.—This paper contains a brief series of experiments performed by Professors Bischoff and Jolly, of which the results corroborate entirely the conclusions drawn by most other physiologists from the facts already known. They establish, first, that even the most delicate galvanometers can detect no current of electricity in the nerves; although they are such bad conductors of electricity, that its passage through them is not discernible even when its force is such as would act on very coarse galvanometers. These two facts together are conclusive against the existence of the supposed natural currents, since if they existed in such bad conductors, it is impossible but that the electrical tension would be sufficient to affect the galvanometer. But on the other hand, it is clearly proved that the nerves themselves are the most delicate of galvanometers, being so irritable to the electrical stimulus that muscular contractions are excited by a current too weak to be detected by the ordinary galvanometer-needle. Other experiments are alluded to which seem to prove that there is no free electricity in either the brain or spinal cord.—*British and Foreign Med. Review, from Müller's Archiv.*

*On the Circulation in the Infusoria.* By Dr. Erdl of Munich.—The author in a letter to Professor Müller says, "I have now very often seen and shown to my friends about here a kind of circulation in the infusoria, a phenomenon so remarkable that I cannot but wonder that it is not mentioned by any microscopic observer. I find it most distinctly in the Bursaria vernalis, whose abdomen, you know, appears to be quite full of green globules. Of these globules, those which lie near the periphery of the animal are incessantly moving (whether the animal itself be still or not) in an elliptic current upwards and downwards. In this current three or four globules always lie close by one another and move together with the stream. It has no relation whatever to the vivid ciliary motion that is constantly going on on the outer surface."—*Ibid, from Müller's Archiv. Heft* iii. 1841.

*On the Functions of the Anterior and Posterior Columns of the Spinal Cord.* By Dr. Kuerschner.—The experiments here related, to establish that the anterior and posterior columns are respectively and exclusively motor and sensitive, are simple and founded on the well known phenomena of reflex action.

The facts that individual muscles can be voluntarily moved, and that isolated sensitive impressions can be perceived, prove that the nervous principle is conducted along the spinal cord according to the same laws as it is along the nerves themselves. Reflex motions, therefore, cannot take place when the supposed sensitive columns are stimulated at the top of a divided cord, for the sensitive fibres cannot conduct centrifugally, nor can any motions take place in consequence of stimulating those columns unless they contain motor filaments. But in several experiments, chiefly performed on decapitated frogs, no motions whatever were excited by stimulating the upper part of the posterior columns, though by the slightest touch of the anterior, severe convulsions were produced. The same result was obtained in whatever way the experiment was modified, so that there can be little doubt that there are no fibres in the posterior columns which are capable of conveying impressions (directly) to muscles.

The converse, namely, that there are no fibres in the anterior columns capable of conveying sensitive impressions, was proved in a similar manner. Thus, reflex motions occur only with the assistance of the centripetal nerves; and therefore when the posterior roots of the nerves of one of the legs of a frog are divided, irritation of the skin of that leg should cease to excite motion. And this in reality occurs; whenever the posterior columns, or roots of the nerves of a limb are destroyed, all reflex motion on irritating its skin is put an end to, although irritation of the anterior columns or roots still produces active muscular contractions.—*Ibid, from Müller's Archiv.* 1841. *Heft* i, p. 15.

*Operations of Laryngotomy.* By M. GUYON.—In a paper on the operations performed with the French army in Africa, M. Guyon states that laryngotomy was twice performed at Bone by M. Génin in croup, "*angines croupales,*" but it did not retard death. M. Vital performed the same operation at Constantine for laryngitis, the symptoms of which ceased after its performance. The wound cicatrized, but the same symptoms returned as before, and the patient died six weeks after he had undergone the operation. On post-mortem examination, the mucous membrane was found tumefied so as to obstruct the opening of the larynx. The same operator, at Algiers, performed the same operation to remove a leech from the larynx, and it was crowned with success. M. Guyon states that he and M. Méardi have seen three cases in which a leech had got into the larynx, but they were removed without a necessity for laryngotomy.—*Ibid, from Gazette Médicale de Paris. Avril* 3, 1841.

*Retroversion of the Unimpregnated Uterus.* By Dr. ALKEN, of Bergheim.—A young woman, twenty-six years of age, accustomed to hard work, found herself suffering one day from difficulty in passing her urine and fæces; and these gradually increased till, after fourteen days, there was complete retention of urine and of fæces. When the author was called in he found the patient with a pale fallen countenance, cold extremities, a small, rapid, jarring pulse, hurried respiration, insatiable thirst for cold water, hiccup, vomiting, &c. The abdomen was very tense, the urinary bladder distended up to the umbilicus, and every movement of the abdomen and the slightest touch extremely painful. Examination detected a complete retroversion of the uterus, so that its vaginal portion was immoveably fixed against the pubes, and its fundus was thrust deep into the pelvis. The patient had been in this state for ten hours. The urine was with difficulty drawn off by the catheter, and after bleeding and the warm bath, an attempt was made to reduce the uterus to its right position by pressing it in opposite directions through the medium of the vagina and the rectum. After the efforts had been continued an hour the uterus returned to its place. The replacement was perfect; but on the following day the retroversion again occurred after some exertion. It was again reduced with much greater facility than before, and by observing the horizontal posture for nearly three weeks, it was prevented from again returning. The patient was watched for several months, and it was clearly determined that at the time of the retroversion the uterus was in the unimpregnated state.—*Ibid, from Casper's Wochenschrift. April* 3, 1841.

*Extraction of a Foreign Body implanted in the Uterus.* By M. MAISONNEUVE, of the Hospital St. Louis.—This patient was thirty years of age on her admission to the hospital, Sept. 14, 1840. At the age of twenty-eight she was in good health, became pregnant, and in the fifth month of gestation states that she miscarried and suffered from severe metro-peritonitis. She was obliged to enter the hospital La Pitié soon after she had begun to leave her room, where the surgeons diagnosticated metritis with hypertrophy of the anterior surface of the uterus. Various means were persevered in without effect, and when she came under the care of M. Maisonneuve her general powers were enfeebled, digestion bad, hectic at night, and she had dull continued pains in the loins and hypogastrium, which latter region was occupied by an irregular hard tumour, slightly painful on pressure, which filled the pelvis and extended into the iliac fossa. The os uteri permitted the entrance of the finger, but the body and neck of the organ were lost in an irregular, hard, and absolutely immoveable mass. Examined by the speculum the vagina appeared to be in the normal state, and the only unusual appearance was abnormal patency of the os uteri, which permitted the surgeon to see something whitish. He passed a stylet to discover the nature of this substance, and was astonished to find that he could circumscribe the unknown object by passing the stylet before and behind it. It adhered to the lips of the os uteri on all sides.

Persuaded that this was a foreign body implanted in the walls of the uterus, M. Maisonneuve first endeavoured to divide it with scissors but could not. He then placed one beak of a pair of long polypus forceps behind and the other before it, and by careful traction removed, without causing much pain, a piece of wooden stick, 122 millimeters in length, pointed at one extremity and bent at the other. Looking to her account of the case, it appeared highly probable that this stick had been broken in the uterus during criminal efforts to produce abortion, and this opinion has been since confirmed.

The operation was followed by a return of abdominal and lumbar pains, and great febrile reaction, which disappeared in about eight days under general bleeding, baths, and several applications of leeches. But there remained a tumour in the pelvis which probably resulted from chronic adhesions of the uterus, bladder, and rectum, with some of the intestines. However by the 1st of January, 1841, the tumour had greatly diminished and the general health was much improved.—*Ibid, from Gazette Médicale de Paris. Avril* 3, 1841.

*Experiments on Uterine Injections.* By M. HOURMANN.—*1st series.* Injections of the uterus separated from the body. Eleven experiments were made. Five times the liquid traversed the fallopian tubes and was effused into the peritoneum; in the other six the tubes were not distended, or but slightly.

*2d series.* Injections of the uterus *in situ.* In five experiments, the liquid passed through the tubes.

*3d series.* Injections of the uterus *in situ* some days after delivery. Injections passed with force from large syringes never penetrated the tubes. The mucous membrane of the uterus and tubes was generally tumefied and red. Four of these experiments were made by M. Danyau.

Among the six cases of non-penetration in the first series, three were in females lately delivered. These seven cases, then, show that uterine injections may be employed without danger after accouchement.—*Ibid, from Gazette Médicale de Paris. Février* 20, 1841.

---

*On the Microscopic Characters of healthy Milk.* By Pr. Von D'Outrepont, of Würzburg.—He has recently put to the test some of M. Donné's statements with reference to the characteristics of healthy milk, and has arrived at somewhat different conclusions, though he fully confirms Donné's statements with reference to the difference of the corpuscles in the colostrum and milk.

Professor Von D'Outrepont found that in the greater number of instances the peculiar granular bodies of the colostrum (*corps granuleux*) disappeared on the third day after delivery and not on the sixth or tenth as stated by Donné. Even in those cases, however, in which they could still be detected on the tenth or twelfth day, the milk produced no injurious effects on the infant; nor did it indeed in some instances in which the milk retained the characters of colostrum so long as a month after delivery. The milk of a female labouring under severe metro-peritonitis presented the characters of true milk, not of colostrum. That likewise from the left breast of a person whose right breast was in a state of suppuration presented all the characters of healthy milk, though pus-globules were mixed with the milk in the other breast. In two instances where the breasts became inflamed without suppurating, the milk continued to present all characters of the healthy secretion and did not contain any of the granular bodies of the colostrum.

Professor D'Outrepont had the opportunity of examining the milk of a woman who, after having suckled her third child for some months, began to menstruate regularly. During the flow of the menses the child became indisposed to suck and suffered from vomiting, but recovered its health immediately on their cessation. During menstruation the milk possessed all the characters of colostrum, while at other times its appearance was precisely that of healthy milk. The secretion from the breasts of a woman who had never been pregnant presented all the peculiarities of colostrum; that contained in the breasts of another woman who never suckled her children, though the glands were always full except during pregnancy, differed in no respect from healthy milk.—*Ibid, from Neue Zeitschrift für Geberts-kunde. Bd.* x. *Heft* i.

---

*On the Chemical Analysis of the Blood in its morbid conditions.* By Dr. Louis Mandl.—The object of this series of papers is to point out the defects and fallacies of the present modes of analyzing the blood, and especially of estimating the *quantities* of its several principal constituents.

1. Three methods have been most commonly employed to separate the fibrine, but all are fallacious. The first consists in squeezing the clot of blood in linen, so as to get rid of its serum, and then repeatedly washing the remainder. Now the *clot* of blood has by no means always the same characters; some clots are hard and dense, and can only with difficulty be broken up, and these may certainly have all their fibrine, which forms compact stringy masses, retained within the linen. But others are soft and friable, and when these are broken up and squeezed, numberless particles of fibrine pass through the fine meshes of the linen, and what remains is but a small portion of what really existed in the blood. Independently therefore of the varying effects of more or less force in pressing the clot, and of the coarseness or fineness of the linen used, it is impossible that by this method, (which was generally employed by M. Denis,) any just idea should be formed of the respective quantities of fibrine in coagula whose physical qualities are different; for in all soft coagula, the fibrine breaks up into pieces small enough to pass through the linen with the serum and colouring matter.

The second method, by stirring the blood, is not less defective. Besides that, even under the best of circumstances, it is not so easy to stir the blood as to get all its fibrine in shreds, here again as in the preceding case the fibrine may be in such a state as to render its separation impossible. In blood mixed with pus for example, it forms in coagulating particles which are either so small that they can hardly be seen, or pieces which stick to the walls of the vessel, or swim in the rest of the blood, and cannot be possibly separated from it. And in many other circumstances a similar condition exists in a less degree, so that this method can be employed only in cases in which the fibrine is disposed to coagulate firmly and in large masses, and has not its tendency to become solid, interfered with by the presence of purulent, or saline, or other substances in excess in the blood.

The method of Berzelius, that of compressing slices of the coagulum in blotting paper, and when they have ceased to give out moisture washing them till they are colourless, is open to different but almost equal objections.

In washing the remains of the coagulum one of two things will happen, either the blood-globules that are retained in it will be washed out entire, and then they will carry with them small pieces of the fibrine itself, or else their fluid contents and their colouring matter only will be removed, and their own membranes and those of their nuclei (which cannot be chemically distinguished from fibrine) will remain. In the former case the quantity of fibrine will appear less than it really was; in the latter it will seem more, perhaps much more; nor can any dexterity of manipulation avoid these errors. Besides from one kind of clot, (the firm one of inflammatory blood,) it is almost impossible to wash away all the globules without breaking it up; and from others (the softer kinds formed in typhoid fever, &c.) the fibrine separates sometimes in particles almost as small as the globules themselves, and cannot be retained on a filter; numberless particles of it may be detected with the microscope in each portion of water that passes through in the process of washing, and many more when (as is commonly the case) the fibrine is washed, not on a filter, but in a vessel of water.

In whatever method it be obtained, the fibrine is subsequently macerated for a considerable time in water, in which, if the process be contained for twelve or more hours, as it commonly is, decomposition takes place, the water becoming turbid, and the fibrine gelatinous. And after this it has to be dried, an operation requiring great delicacy and judgment to ensure that at the end of it water shall not be still retained in the organic substance which so readily absorbs it.

And after all it is very doubtful whether under the best of circumstances the fibrine extracted from the blood is all that it really contained, for none of the analyses yet published take into account that which in all probability exists in the envelopes and contents of the globules, and which may well amount to more than there is in the *liquor sanguinis*. For several things are quite inexplicable on the supposition that as commonly stated there are only three parts of fibrine in 1000 of blood; as for example, the immense quantities effused in false membranes, amounting in some cases to more than the whole mass of blood is said to contain, and many others.

If it be said that in spite of all these sources of fallacy the analyses of different chemists closely accord; reference need only be made to those analyses, and it will appear that the quantities of fibrine said to exist vary from 0.75 (Berzelius) to 4.96 (Müller) parts in 1000. Besides, these analyses were made on healthy specimens of blood; the difficulties are greatly increased when it is in a morbid state, when the determination of its quantity of fibrine is most important.

2. The composition of the globules is as yet rather uncertain, but the author has little doubt that their contents are in part fibrine, by the coagulation of which the nucleus is formed; and he thinks that their membranes are also fibrine, coloured with hematosine. Now, as already stated, this fibrine within the globules has not in any analysis hitherto published been taken notice of; nor is it right to estimate its quantity from that of the albumen (as it has been supposed to be) which chemists have obtained from the globules: for it is difficult in any case to collect all the globules in a sample of blood; a number of them always float in the serum, and the more the softer the clot is, so that in many cases the serum itself is of a deep red; and moreover, in the ordinary plans of treatment, the clot is never obtained completely free from serum, and therefore from a greater quantity of albuminous matter than properly belongs to its globules. In short there is not at present any method known by which the globules can be all removed from a portion of blood, and obtained separately from its serum and other principles; and there cannot therefore be any certainty in the calculations of their weight or of the quantity of fibrine or albumen which they contain.

3. The fallacies in the analysis of the serum depend chiefly on the quantity of globules and of portions of fibrine that may be suspended in it. These in the healthy state of the blood are unimportant, but in its diseased states they may give rise to considerable errors.

In the next division of his subject the author treats of the coagulation of the blood, and the formation of the buffy coat, of the chemical nature of which, he rightly says, nothing is at present known. [But under this head we find nothing worthy of being abstracted.]

In the application of his objections, M. Mandl takes two classes of cases, namely, those in which the blood is said to be poor in fibrine, including scurvy, putrid fevers, typhoid fevers, &c., and those in which it is described as rich in globules. For the first the quantity of fibrine will appear small in all cases in which a firm clot does not form, that is, not only when there is really little fibrine but when that which does exist, after coagulating, does not contract and form a dense hard clot. And this may depend on the presence of pus or other morbid fluids in the blood, or, still more probably, on an excess of alkaline salts. The latter are known to exist in excess in scurvy, and perhaps may be found so in the other diseases in which the blood has been less carefully analysed. At any rate, it is certain that we have at present no means of ascertaining the real quantity of fibrine in a soft clot, and therefore no right to assume a deficiency of it in any cases till the softness of the clot is proved not to depend on some other cause than that deficiency. Similar difficulties attend the appreciation of the true quantity of globules; and hence the diversities of the statements of authors respecting their increase or decrease in different diseases.

The last part of these papers contains general reflections on the application of the chemical researches on the blood to pathology. [But in this also the remarks are trivial. Indeed, the author has evidently much more skill in finding fault than in improving defects. We have ourselves long thought that in the matter of the blood, physiologists have too implicitly received the statements of chemists; and we hope that the brief abstract we have given of M. Mandl's papers, though they do no more than point out the numerous and unavoidable fallacies of these analyses, will engender caution in drawing conclusions from such evidently insecure premises.—*Ibid, from Archiv. Gén. de Méd.*

---

*Account of the Petersburgh Foundling Hospital, and Establishment for bringing up Children.* By J. G. Kohl.—This establishment was founded by the Empress Catharine in 1770. Its extent was of course at first but limited, and even in 1790 there were only three hundred children in it. But since the commencement of the present century the number of its inmates has very greatly increased, and has varied at different times, from one thousand to five, ten, or even five and twenty thousand. In 1837 there were no less than 25,600 young people in this gigantic establishment, and the number of children admitted into it of late years has been from 1828 to 1829, between 3 and 4000; from 1830 to 1833, between 4 and 5000; and from 1835 to 1837, between 5 and 7000.

The admission of children is perfectly unrestricted: every one that is brought is taken in, and without further ado received into the establishment, and the government, so far from setting a limit to the admissions, has rather taken care to provide for the wants of the charity with extraordinary liberality. The original foundation by Catharine was, in proportion to the present means of the Institution, extremely small; but it has been increased by munificent presents from private individuals, and still larger gifts from Alexander, Paul, and Nicholas; and it is now one of the richest charities in Russia, and has many millions (of rubles) invested in houses. To these sources of income Alexander added the revenue derived from the manufacture of cards, and that from the *Lombard* (or general establishment for pawning, *Mont de Piété*,) which, in consequence of the great fluctuations in private property in Petersburg, is an establishment of enormous extent. Thus it is that every year six or seven hundred millions of rubles are available for the Foundling-Hospital, and pass through the hands of its director. The maintenance of the whole establishment now costs 5,200,000 rubies a year; in 1837, two millions was spent in building, and 300,000 in the erection of a church for the inmates.

The principal establishments are at Petersburg and at Gatschina, but their benefits are extended over the whole neighbourhood of Petersburg. In the latter city are the chief buildings for the reception of children of both sexes, and for the care of them during the first six weeks; after which time, when they can bear removal, they are sent to people in the villages and towns within a circuit of 130 wersts from the city, with whom they remain till they are six years old. At that age the girls are brought back to Petersburg for further education, and the boys are sent for the same purpose to the establishment at Gatschina. When their education is completed, they are all permitted to leave the Institution, and are left perfectly free to follow their own choice of an occupation, or are sent to the business for which, according to their capacities, they have been prepared.

Six or seven hundred nurses are kept at the Institution, and twelve physicians, mostly Germans, officiate in the hospitals attached to it, and have the charge of examining and frequently visiting the children that are sent into the provinces. A lying-in hospital is also attached to the Foundling, and is based on the most liberal principles, every one that wishes being admitted without restriction. Complete secrecy, however, is maintained over this part of the establishment, and it is open to none but those who are actually attached to it.

At the Foundling the door of the little receiving room is open day and night throughout the year, and an inspectress, with several female assistants, is constantly in attendance. A thick book is kept in which the children, amounting to fifteen or twenty daily, are entered. The only question asked on receiving each of them is, whether it has been baptized, and whether it has a name. If this be the case, the child is entered in its own name; but if not, it is entered with a certain number attached to it. The women come in the greatest numbers at the close of the evening, with their children wrapped in cloths, and their attendance is more numerous in fine than in bad weather, and in summer than in winter, but most of all in the spring. We were at the Institution at one o'clock in the day, and at that time seven new inmates had been received, whose numbers we saw entered in fresh ink, 2310 to 2317. It not unfrequently happens that when the mother undoes the clothes, she finds her child dead; they are then not received, but are sent to be examined by the police. The living children, however, are at once received, without reference to their origin; they are all baptized and admitted into the true church, and after six weeks are sent into the provinces. A fourth of them, however, die within these six weeks, and half of those that are sent away die within the next six years, so that only about a third of those that are received survive their sixth year, while that of the children brought up at their own homes fully half survive that time. A great proportion of this heavy mortality depends on the great distances through which they are carried to and from the establishment, for many of them come from remote parts of Russia, (indeed from all parts except those from which the superfluous children are sent to Moscow,) and many are half dead before they arrive. In 1836, for example, there arrived on one day a child from Kischeneff, in Bessarabia, and another from Tobolsk, in Siberia, both of which places are about 250 miles from Petersburg. How many therefore must die even before they arrive at their destination!—*Ibid, from Casper's Wochenschrift.*

---

*Case of Intestinal Invagination in the Cow, cured by Gastrotomy.* By M. Bouley.—At the sitting of the Royal Academy of Medicine, March 16, 1841, M. Bouley related a case of intestinal invagination cured by gastrotomy in a heifer. The animal had suffered from colic, and on the third day the symptoms were such as led the veterinary surgeon to suspect internal strangulation. He therefore introduced his arm into the rectum and felt a tumour in the iliac region, which confirmed his diagnosis. The seventh day the symptoms persisting, he performed gastrotomy, guided by the tumour in the iliac fossa, which he then found to consist of five or six pouches of intestine, agglutinated together by effusion from the peritoneum. The operator broke up their adhesions, and withdrew the invaginated portions of intestine by gentle tractor. These portions were perfectly healthy. The opening in the abdominal parietes was united by a suture, and a perfect cure was expected. It may be questioned whether a similar operation would be successful in the horse, as this animal is more liable to peritonitis than the bovine race.—*Ibid., from Gaz. Med.*

---

*On the Functions of the Roots of the Nerves.* By M. Longet.—Last year M. Longet addressed the Royal Academy of Medicine, deducing from certain experiments the inference that the anterior or motor root of the spinal nerves was endowed in a certain degree with the faculty of sensation, and that it owed this faculty, not to its relations with the antero-lateral column of the spinal marrow, but those which it has, by means of the spinal ganglion, with the corresponding posterior root. M. Longet has since repeated and modified these experiments, and finds, after trials upon seventeen dogs, and upon the roots of ten nerves in each dog, (thus making 170 experiments,) that uniformly, the anterior roots and the corresponding columns of the spinal marrow are insensible to every kind of mechanical irritation, while the posterior roots and the posterior spinal columns are always exceedingly sensitive. "But," he adds, "in applying alternately the two poles of a voltaic pile of twenty couples to the two roots under similar circumstances, I excited the most violent convulsions in acting on the anterior roots, while not the slightest trace of convulsions was manifested in experimenting on the posterior roots." In all these experiments the roots were isolated by plates of glass. Whether mechanical irritation, then, or galvanism be employed, the phenomena are so uniform and so evident, that we cannot doubt that the anterior roots are exclusively motor, and the posterior exclusively sensitive.

Galvanism may pass from the anterior column of one side to that of the opposite side, through the medium of the anterior white commissure which unites them; but it is worthy of remark, that it never passes from the posterior to the antero-lateral column through the medium of the posterior corner of gray substance which completely separates these two columns. The gray substance, then, appears to be a bad conductor of electricity.—*Ibid., from Jour. des Con. Medico-Chir.*

---

*On the Impropriety of Dividing Muscles of the back in lateral Curvatures of the Spine.* By M. Bouvier.—After numerous experiments, M. Bouvier concludes:

1. That the section of the sacro-lumbalis, longissimus dorsi, spino-transverse muscles, &c. is not immediately followed by any diminution of spinal curvature.
2. The changes which the curves undergo during the succeeding mechanical treatment are exactly identical with the changes produced by this treatment alone, when it has not been preceded by the section of the muscles.
3. The space of time necessary to obtain these changes is the same whether we have recourse to orthopedic means alone, or practise also section of the muscles.
4. In a word, dorso-lumbar tenotomy has no kind of influence in remedying lateral deviation of the spine, properly so called.

M. Bouvier further concludes: 1. That the majority of lateral curvatures of the spine are not owing to muscular contraction; and, 2. That etiology, pathological anatomy, and clinical experiments proscribe the section of the muscles of the back in the treatment of these curvatures.—*Ibid., from Ibid.*

---

*On the respective Functions of the Upper and Lower Halves of the Spinal Cord, and their relation to the Extensor and Flexor Muscles of the Limbs.* By Ed. Englehardt.—If, after cutting off the head of a frog, a wire be pushed slowly from above downwards through the vertebral canal, the two thighs are drawn forcibly to the abdomen, and the feet are made to meet above, and are pressed against the wire; in short, motions of the legs take place which have exactly the appearance of being intended to put away the wire. If the wire be now carried farther into the spinal marrow, the same motions still, for a time, continue to be produced, though less forcibly than before; but, when it has been pushed to about the middle of the vertebral column, they assume all at once a different character, and the legs are no longer drawn up to the abdomen, but are forcibly thrust away from it. The deeper the wire is passed, the more powerful do these movements of extension become; nor do they cease till the very last portion of the spinal marrow is destroyed.

To determine more exactly the spot at which irritation of the spinal marrow ceased to produce flexion, and brought on extension of the legs, I instituted the following experiments on a number of frogs: I cut off the head with scissors; and at the instant the legs were every time drawn up forcibly towards the abdomen, and the feet pushed against the scissors. I then removed the abdominal and thoracic organs, laid the trunk on its back on a smooth board, placed the legs in a half-flexed position, and cut through portions of the spinal marrow at each vertebra successively, proceeding regularly from above downwards. The legs were every time, as soon as the motions produced in them by each section had ceased, again placed in the half-bent posture before another section was made. The result was as follows: From the first to the fourth vertebra, the sections of the cord produced flexions of the thighs and stretching out of the feet over the upper part of the trunk. The more there was cut off from the cord, the weaker were these movements; and, when the sections came to be made at the part between the fourth and fifth vertebra, the legs were no longer drawn up, but movements of extension were produced in them, which increased in force in proportion as the sections were made nearer to the os coccygis. When the whole spinal marrow was thus removed, and portions were at the same time cut off from the three nerves which compose the sacral plexus, the result was, that only movements of extension of the legs were produced; and these continued till the section (carried onwards by degrees) came to be made at the part where the plexus divides into the ischiatic and the crural nerves.

To see whether the anterior limbs were similarly influenced by stimulating particular portions of the spinal marrow, I modified the experiment in the following manner: after cutting off the head, (by which the fore-legs were thrown into violent contractions,) I removed the abdominal and thoracic organs, without injuring the walls of the chest or the muscles of the shoulder attached to them. I then divided the vertebral column between the eighth vertebra and the sacral vertebra, and pushed a wire from below into the vertebral canal. The result was, that while the lower half of the spinal marrow was being destroyed, the anterior limbs were extended; but as soon as the wire reached the upper half of the cord they were flexed.

To test the influence of division of the upper and lower halves of the cord respectively on the reflex motions, I repeated the above experiments on several other frogs; and after replacing the legs in the half-bent position, as soon as the motions produced in them by the division of each vertebra and the cord within it had ceased, I stimulated the toes of one one of the feet by pinching them with forceps. I now observed, that so long as the cord was not cut away by the successive sections, so far as the space between the fourth and fifth vertebræ, movements of flexion only were produced in the irritated leg, and in that opposite to it.—But if the division were made at *this* spot, then irritation of the toes of one foot no longer produced flexion either in the corresponding or the opposite leg, but in several cases excited extension, which, however, was weaker than when the marrow itself was directly stimulated. As soon as the spinal marrow was divided between the fifth and sixth vertebræ, the reflex motions ceased, though direct stimulus of the marrow even down to the sacral vertebra still produced the same extension of the hind-legs as in the first-mentioned experiments.

Now, as the vertebral column of the frog consists of eight free vertebræ and one sacral, and division of the spinal marrow from the medulla oblongata down to the fourth vertebra produces flexion of the limbs, and from the fifth vertebra to the end of the column produces extension; and since, moreover, the experiments on the alterations of the reflex motions, according as the marrow is divided in its upper or in its lower part, produce a result which exactly agrees with these, it appears to follow that there is a determinate antagonism between the functions of the upper and the lower half respectively.—*Ibid, from Müller's Archiv.*

---

*On Scorbutic Pericarditis, and Pleuritis, and the Cure by Paracentesis.* By Dr. Karawagen, of Kronstadt.—The scurvy epidemic which prevailed among the sailors at Kronstadt during the months of May, June and July, in 1839, was particularly marked by the effusions of blood into the cavities of the chest which are known by the names of *pleuritis* and *pericarditis scorbutica.* The common sign of both these effusions, which commonly take place very rapidly, is an extreme difficulty of respiration. In the pericarditis the heart's sounds appear faint and remote, and percussion yields a dull sound over an unnaturally wide extent. In the pleuritis, only a weak respiratory murmur can be heard on the affected side; percussion is dull, and the respiration is short and hurried. There are also present slight fever and most of the ordinary signs of severe scurvy, which indeed always precede by one or more days the more dangerous symptoms of the thoracic effusions. When the latter come on, the condition of the patient is at once one of imminent danger, and the pericardial hæmorrhage is rarely survived beyond the third day.

In the examinations of sixty patients who died of scurvy, the author found that thirty had this kind of pericarditis, twenty-two had pleuritis, two pleuritis and pericarditis together, and two peritonitis. In the pericarditis the pericardium appears three times as large as natural. It contains a bloody, dark-red exudation, which may amount to as much as four or five pints; so that it might be suspected that some vessel had been ruptured, though on examination none such can be found. The heart itself appears three times as small as usual; its surface, as well as the lining of the pericardium, is covered with a layer of red mould-like substance that may be scraped off with a knife; and beneath this the substance of the heart is soft and fatty. The endocardium is red, probably from imbibition; the ventricles are greatly compressed, and always empty; the left lung is compressed and bloodless. When there has been pleuritis, ten or twelve pints of bloody fluid are found in the pleuræ, whose surface is similarly covered by a layer of mould-like substance, and is very red and vascular. In peritonitis the appearances are similar, but the quantity of fluid amounted (in the cases examined) to from thirty to thirty-five pints.—Once, in a case in which the patient died with symptoms of apoplexy, a similar effusion was found in the cavity of the skull. Sometimes the bones and fibrous tissues were affected with scurvy, and this was especially the case with the ribs, whose ends were found separated from their cartilages, moving on them with a kind of crepitation, and looking as if they had been eaten away by caries.

In despair of any other remedy, the author determined on tapping the serous cavities into which the hæmorrhage had taken place. He performed this operation six times: on four patients with pleuritis, and on two with pericarditis; one of the last recovered completely, and all were immediately relieved and had their lives prolonged. The case of the man whose life was saved, and who was the only patient that recovered after pericardial effusion had evidently taken place, is related at length. He appears to have been in extreme peril when the operation was performed; but directly after three pints and a half of fluid had been extracted he felt much relieved, though a quantity of air had passed into the pericardium through the trocar; and he gradually recovered under the use of the ordinary antiscorbutic and some other means. Five months after the operation he might be considered quite well.—*Ibid, from Medicinische Zeitung.*

---

*Researches on the Anatomy of the Aponeuroses and Muscles of the Eye, in relation with the cure of Strabismus.* By M. Bonnet, Surgeon to the Hôtel-Dieu at Lyons.—These researches conduce to the scientific explanation of the persistence of the action of the orbital muscles, after the section of their anterior part in the operation for the cure of strabismus; they show the method to be followed in this operation, and throw some light on the movements of the eye and eyelids, considered in a normal state.

The eye is not in contact with the fatty matter of the orbit, as anatomists have stated; it is separated from it by a fibrous capsule, in which it moves with facility. This capsule, concave and open within, is inserted on the anterior extremity of the optic nerve, around the two posterior thirds of the eye without being in contact with them, and terminates on the eyelids on which it is prolonged. The straight and oblique muscles traverse it to reach the eye and contract, with it, intimate adhesions. They have thus two insertions, the one into the sclerotic, the other to the fibrous capsule, and they cannot move one without transmitting to the other all the movements they execute. This has not been hitherto noticed by anatomists, and we shall trace their operation on the movements of the eye and eyelids.

We know that when one of the muscles of the eye has been cut in the operation for strabismus, the increased action which caused the disease immediately ceases, and the movements attributed to the divided muscle are executed as in the healthy state. The explanation of these effects, to be satisfactory, should apply to any of the muscles of the eye indifferently, for after division of any of them the persistence of their function is observed. This cannot be attributed to any phenomenon, which requires, like cicatrization, a work of many days, for the motions effected by the divided muscles are manifested immediately after their section has been made. The anatomical explanation is founded on the fact that the muscles of the eye are inserted both into the sclerotic and the fibrous capsule, and the first of these insertions alone is cut in the operation for strabismus. The second remains entire; the muscle continues to act on the fibrous capsule, and by this medium transmits to the eye these contractions simply weakened.

This double insertion of the muscles of the eye, and the adhesions of this organ to the fibrous capsule explain, it is true, the persistence of the action of the muscles after their division, and indicate the conditions of this persistence, but they do not lead to the knowledge of the method to be adopted in operating for strabismus. But this knowledge is gained in part, at least, from the dispositions of a fibrous membrane immediately applied over the whole external surface of the sclerotic. This membrane, altogether distinct from the capsule before described, is confounded with the fibrous sheaths of the muscles, and serves to unite them to each other, forming an immediate layer between the conjunctiva and sclerotic. This must be traversed in the operation, and when by its section we have reached the lax cellular tissue which unites it to the eye, the stylet glides without obstacle behind the sheath of the muscles, which, with their aponeurosis, can then be divided certainly and entirely. The knowledge of this membrane gives an astonishing facility to the section of the muscles, both in the living and dead subject, and is as important in the operation for strabismus as that of the sheath of the arteries in the ligature of these vessels.

If it is demanded how the beautiful harmony which always exists between the elevation and depression of the eyelids and similar motions of the eye; or what muscle depresses the lower eyelid; questions hitherto unresolved: their phenomena are readily comprehended when we know that the tarsal cartilages are the continuation of the fibrous capsule into which the latter is inserted, and which conveys the motions of the levator and depressor muscles of the eye. These muscles cannot contract without acting at the same time on both eye and eyelids, and the cause of this simultaneous action is simply anatomical, for we cannot move these muscles in the dead body after having denuded their posterior half, but the eyelids move at the same time and in the same relation with the globe of the eye.—*Ibid, from Bul. Gén. de Thérapeutique.*

---

*Removal of a large part of the Rectum affected with Scirrhus.* By Dr. Francescc Rizzoli, of Bologna.—The patient, Paula Porticelli, was about forty-eight years old when the catamenial discharge permanently disappeared.—Numerous hæmorrhoidal engorgements supervened, with a sense of pain and weight along the internal part of the sacrum, and constipation, which at length became so obstinate that the patient passed thirteen days without alvine evacuation. There was a discoloured fetid exudation from the intestine, and the fæces which came away were in the form of small cylinders. On examining the part scirrhous tubercles were observed, and on passing the finger into the rectum, hard and fungous carcinomata projected, which, about three inches and a half up, were disposed circularly, forming a kind of ring, within which the end of the finger could be introduced with difficulty. An operation being determined on, the patient was placed in bed on the left side, with the thighs bent at right angles with the body, and the nates held apart by an assistant. The operator, with a curved bistoury curved forwards, made a semicircular incision on each side of the anus, which met posteriorly at the coccyx and anteriorly at the perineum; and when the integuments and adipose tissue were divided, he detached the inferior portion of the rectum, taking care to preserve the sphincters. The right index-finger was then introduced above the scirrhous ring, and used to draw it down as far as possible; then the interior portion of the intestine being held to the right and left sides by two assistants with Museaux forceps, and at the same time drawn lower down, Dr. Rizzoli insulated it from the surrounding cellular tissue by the index-finger of the left hand, except where it is connected with the vagina, in which place the union being more intimate and firmer, it was necessary to detach it, partly with the bistoury and partly with a spatula; the finger was then again passed beyond the scirrhous ring, which being used as a guide, by means of a pair of long forceps the intestine was removed by incising it all round, taking care that the edge of the instrument should reach the sound part. After exploring and removing all the diseased parts that remained behind, the bleeding arteries were secured by ligature and torsion, and the venous hæmorrhage arrested by means of plugging, and the proper dressings applied. The operation was succeeded in a few hours by fever, which was relieved on the fourth day by five bleedings, and the catheter had to be applied twice. The dressings were changed on the third day, when the patient was again bled, and caster oil given, which procucured a copious evacuation followed by great relief. After a few days granulation commenced, and the alvine and vesical evacuations were regular. The patint was attacked with phlebitis on about the thirteenth day, which appeared in both legs, and which gave way to cataplasms and bandaging. Afterwards, the new portion of intestine having a tendency to contract, mechanical dilatation was applied, and the patient continued to live free from any inconvenience and without further medical treatment.—*Ibid, from Bul. delle Scienze Med. di Bologna.*

---

*On the Formation of Artificial Anus in Adults, for the Relief of Retention of the Fæces.* By John Erichsen, Esq.—The following are the cases which require the formation of an artificial anus:

1st. Stercoral tympanitis produced by obstruction of the rectum, or of the sigmoid flexure of the colon. Whether this obstruction be the consequence of disease of the intestine itself, or of any of the neighbouring parts, provided we cannot overcome the obstacle from below, and that life consequently is in danger, an artificial anus must be established.

2d. Simple retention of the fæces producing a stercoral tympanitis, which cannot be relieved, and which endangers life.

3d. Scirrhous affections of the rectum, as soon as there is much difficulty in defecation. The establishment of an artificial anus is, in these cases, the only means of retarding the progress of the disease.

4th. Imperforate anus, or rather the absence of a portion of the rectum, when the passage cannot be established in its natural situation.

Amussat states that during his practice he has seen at least seven or eight patients die of stercoral tympanitis, in consequence of his not having dared, in accordance with the views of most surgeons, to open the obstructed bowel.

It was whilst attending Broussais, who died of obstruction to the passage of the fæces, produced by a scirrhous disease of the rectum, that Amussat was led to reflect on the best means of relief in similar cases, and he determined upon putting his project, of opening the colon from the left lumbar region, into execution, as soon as he had an opportunity of so doing; and for this he had not to wait long, as two cases soon occurred to him which fully justified the performance of this operation.

Case I.—Madame Dubois, aged 48, of a sanguineo-nervous temperament, had been for several years subject to constipation, her stools being painful, and occurring at intervals of seven or eight days. She has been in the constant habit of using clysters. She always had the appearance of good health, and all her functions were well performed, with the exception of the act of defecation. About the beginning of May, 1839, she was attacked with most obstinate constipation; clysters, baths, and the most powerful purgatives, failed in producing any effect. M. Amussat, who was called in on the 29th, examined the rectum, with a view to remove hardened fæces, which he thought were probably lodged there, but he found the intestine empty. At this time the patient suffered dreadful pains in the bowels, and uttered the most piercing cries, as if she were in labour. There had been no alvine evacuation for twenty-six days, but the desire to go to stool was incessant. Amussat again examined the rectum more carefully than before, and, after a good deal of difficulty, found that, at the upper part of this gut, there existed a round hard tumour, about the size of an orange, and not very moveable; it appeared to be connected with the anterior wall of the sacrum, and completely obstructed all passage through the rectum. The mechanical nature of the obstacle being thus ascertained, it was determined to operate. Accordingly, the 2d of June was fixed for the day of operation, at which Amussat invited me to attend. At this time the patient was labouring under the following symptoms, which closely resembled those of strangulated hernia. She had nausea, with almost constant vomiting and hiccough; the abdomen was painful on pressure, and enormously distended, its circumference being nearly doubled; the face was red and injected; thirst great; anxiety extreme; and she uttered the most piercing cries, calling out for some operation for her relief.

The patient having been laid on her face, with the body inclined somewhat to the right, two or three cushions were placed under the abdomen, so as to raise the left lumbar region. The operation was then performed in the manner that has already been fully described.—When the colon, which was reached without any difficulty, was punctured, an immense quantity of gas and liquid fæces spirted out in a jet; there being sufficient to fill three large wash-hand basins. Injections of warm water were then made in both directions along the intestine, and brought away a large quantity of more consistent fæces, which were covered with a thick mucus: the intestine was then drawn forward by three spring artery forceps, by which the sides of the incision in it had been held open whilst its contents were discharging, and fixed to the sides of the incision in the skin, by means of four points of interrupted suture: only two small anterial branches were divided, the hæmorrhage from which was readily arrested by torsion.* The patient expressed herself greatly relieved immediately after the operation, and said that she felt more comfortable than she had done for several weeks previously. She was then cleaned and put to bed, a poultice having been applied to the wound.

In the after-treatment nothing of consequence occurred; on the 13th of July she was sufficiently well to be able to go into the country. The stools, which were at first glairy, liquid, and abundant, soon became more consistent and regular. The fæces, which are figured, are readily retained by a compress and bandage, so that the patient is not in the least offensive either to herself or to her friends. There is an interesting fact connected with this case, which throws some light upon the physiology of the rectum, namely, that the motions, although figured, and having the same appearances as those which were voided by the natural passage, have little or no fæcal odour.

Five months after the operation the patient was in an extremely good health; her complexion was clear, and she had not the appearance of one suffering from any malignant disease; so that probably the tumour in the pelvis, which appears in every respect to be stationary, is of a simple nature. The appetite is better than before the operation, probably because digestion is more rapid and because the excrementitous matters remain a shorter time in the intestines.

The inconvenience of the artificial anus is very trifling; much less so than would, *à priori*, be supposed: when the patient forces, or makes any effort, the mucous membrane has a tendency to protrude: this, however, can easily be returned, by means of slight pressure exerted by a compress and bandage.

Case II.—Mr. T., ætat. 62, has for several years suffered from painful and difficult defecation, the fæces being very fœtid, and mixed with purulent and ichorous matters. This state having continued for three years, he became exceedingly emaciated, and when the stools came away he felt excessively exhausted and faint.

* I may mention incidentally, that Amussat has informed me that he has employed torsion of the femoral artery seven times in amputations of the thigh; of the axillary once, in amputation of the shoulder-joint, and of the brachial several times, in amputation of the arm, with complete success.

On examination, it was found that he had a carcinomatous affection of the upper part of the rectum, consisting of scirrhous vegetations, arising from the inner surface of that intestine, and nearly blocking up its cavity. The finger could be made to pass into the constricted part, but could not reach its upper termination; below there was a prominent ring, feeling like the neck of the uterus, largely opened and cancerous.

It was determined, in consultation, to attempt to break down the tumor: this was done with long forceps, which crushed and pinched its most prominent part; cold water was then injected, in order to keep down inflammation: this was repeated several times, and a considerable portion of the tumor was thus removed. Cauterizations with caustic potass were then had recourse to, and were repeated several times, at intervals of three or four days; by these means the tumor was diminished to half its original size. However, the patient's condition grew most alarming; he had no stools for twelve days, and then with such violence as to lower him till he fainted. His emaciation was extreme, and the symptoms altogether so serious, that, in order to save him from a speedy death, it became necessary to adopt any resource that surgery might offer. The operation for artificial anus, which had lately been performed on the preceding case with success, suggested itself as presenting a double object—namely, in the first place, to relieve the retention of the fæces, and secondly, to prevent their injurious action on the diseased intestine.

Accordingly, on the 14th of July, 1839, the patient not having had a stool for eight days, the operation was performed. It was begun in the way that has already been described; but when the operator had dissected through the cellulo-adipose tissue covering the peritoneum and intestines, he found that he was too much to the outer side, and that the colon, being strongly retracted upon itself, was concealed under the quadratus lumborum: it thus became necessary to cut across some of the fibres of this muscle, and then, the intestine having been seized with the usual precautions, which have been already described, was incised in the posterior third of its circumference: nothing came away at first but gases, and a few balls of fæcal matter, and it was not until the fourth day after the operation that the contents of the bowels were freely discharged through the artificial anus. As the aperture in the lumbar region evinced some disposition to close, it was necessary to keep it open with tents and sponges. The health of the patient gradually improved, and in a month after the operation he was able to leave for the country. His appearance was at this time greatly changed for the better; his complexion had become clearer, his appetite had returned, and all the functions were well performed; there was no longer any tympanitis, or forced retention of the fæces, regular and figured motion, being passed by the artificial anus. The disease of the rectum had somewhat improved, having lost its granular and vegetative feel: if, however, it should retrogade, it will not, as long as it is confined to the rectum, compromise the life of the patient, but if it extend beyond these limits, of course his condition will be altered.

The opening of the artificial anus is now (four months after the operation) rounded, and the mucous membrane is deeply seated: this opening has a great tendency to contract, but it can readily be dilated by tents and prepared sponges.

It is usually said that an artificial anus is a very disgusting and loathsome infirmity; but it is necessary to distinguish between the different kinds of artificial anus, and also to take into account their situation; for it is evident that an accidental artificial anus, the consequence of a hernia or of a wound, being generally situated in the small intestines, the results of defecation will, in such a case, be very different from those that are observed when an artificial anus is established in the large intestine; so, likewise, an artificial anus, situated towards the front of the body, will be more disgusting than one situated posteriorly. In the two patients whose cases have been just related, a simple bandage and compress were fully sufficient to retain most completely the contents of the bowels, which were solid, and possessed but little odour.

It will be seen by the accompanying table, that eight operations for artificial anus have been performed on the adult. Although Littre had, in 1710, proposed opening the intestines in cases of obstruction of the rectum, it was not until the year 1776 that his proposal was carried out by Pillore, who was the first to perform any operation of this kind. It will also be seen by the table, that four men and four women have been the subjects of operation: in all of them the disease for which the operation was performed was an obstruction of the upper part of the rectum, or of the lower end of the sigmoid flexure of the colon, probably of a scirrhous nature.

With regard to the operative proceedings that have been employed:—Pillore opened the cæcum from the right iliac region. Freer, Pring, and Martland, adopted Littre's method, as modified by Duret, namely, opening the small intestines from the left iliac region. Fine, of Geneva, opened the colon immediately below the umbilicus. Velpeau performed Littre's operation, somewhat modified by himself; and Amussat, in his two cases, opened the colon from behind, in the left lumbar region.

*Table of the Eight Operations for Artificial Anus on Adults.*

| *Date of Operation.* | *Name of Operator.* | *Sex.* | *Age.* | *Nature of the Case.* | *Nature of the Operation.* | *Result.* |
|---|---|---|---|---|---|---|
| 1776 | Pillore. | Man. | — | Stricture of the rectum. | Incision in right iliac region to reach the cæcum. | Death 28 days after the operation. |
| 1797 | Fine. | Woman. | 63 | Schirrus of the rectum. | In the umbilical region. | Death in 3½ months. |
| 1818 | Freer. | Man. | 47 | Stricture of the rectum. | Littre's operation. | Death in 8 days. |
| 1820 | Pring. | Woman. | 64 | Stricture of the rectum. | Littre's. | Was living 5 or 6 months after the operation. |
| 1824 | Martland. | Man. | 44 | Stricture of the rectum. | Littre's. | Was living a year after the operation |
| 1839 | Amussat. | Woman. | 48 | Obstruction of the rectum from a tumor in the pelvis. | Callisen's, modified. | Living. |
| 1839 | Amussat. | Man. | 62 | Schirrus of the rectum. | Callisen's, modified. | Living. |
| 1839 | Velpeau. | Woman. | 70 | Schirrus of the rectum. | Littre's. | Death in 2 days. |

Thus, of the eight operations for artificial anus performed on the adult, we find that in six the peritoneum was opened : of these six cases, three died of peritonitis, in consequence of the operation; whilst the two cases in which the peritoneum was not wounded, have been completely successful. Although these numbers are too small to warrant any general deductions, they are nevertheless highly satisfactory.

The following are some of the conclusions that Amussat comes to :—

1st. The idea of opening the colon in the left lumbar region is not new; but the unsuccessful trials of Callisen and of Duret on the bodies of children had caused it to be rejected.

2d. Trials on the dead body carefully conducted, prove, however, that the operation is practicable, simple and easy of performance, and that it is founded on positive anatomical facts.

3d. The transverse incision which Amussat adopts in preference to the longitudinal one of Callisen facilitates the operation, and allows us to establish the artificial anus somewhat to the side, and not altogether behind. It thus possesses the triple advantage of facilitating the search for the intestine, of allowing the anus to be made a little more to the side, the patient being thus better able to adjust any retentive apparatus that he may employ, and of enabling the surgeon to avoid wounding the peritoneum.

4th. It is of the greatest importance that the intestine, after having been opened, should be drawn well forwards and firmly fixed to the incision in the skin, by means of sutures, so as to prevent effusions into the loose cellular tissue of the wound.

5th. The artificial anus is a much less disgusting infirmity than would *à priori* be supposed.

6th. Reasoning and fact prove incontestibly, that the preference should be given to Callisen's operation as modified by Amussat.—*Med. Gaz.*

*Dr. Watson on the Treatment of Tetanus.*—The *treatment* of tetanus is a mortifying subject. The disease is and has always been a lamentably fatal one. Hippocrates says, *επι τρωματι σπασμοσ επιγενομενος, θνανασιον*, tetanus, supervening on a wound, is morial: and the aphorism holds true, with very few exceptions, in the present day. Almost all the acute and severe traumatic cases are fatal. Hennen declares that he never saw a case of "acute symptomatic tetanus" recover. Dr. Dickson found all curative measures followed by "unqualified disappointment." Mr. Morgan uses these words: "I have never yet seen or heard of an instance of recovery frɔm acute tetanus. Another of Hippocrates' aphorisms is, *ὁκοσοι υπο τετανου ἁλισκονται εκ τεσσαρσιν ἡμερησιν αποληκυνται*; they who are seized with tetanus, die within four days: but he adds, *ην εδ ταυτας διαφογωσιν ὑγιεες γινονται*; if they get over this period they recover. And to this we can only add now, that those who survive the first few days, and ultimately get well, recover in a variety of different ways, and under various modes of treatment. But as to the mode of treatment which is to be preferred, or even as to the real efficacy of any mode, there is much room for doubt. Under every plan of management a vast majority die.

Let us briefly pass in review the principal remedies that have been tried, and enquire what degree of success has followed their employment.

One drug from which much benefit has been hoped for, is *opium*. In some spasmodic disorders it is of unquestionable service. Very large doses of it have been given and borne in tetanus; and some have recovered under its use, and more have died.

It is well known that pain fortifies the nervous system against the peculiar influence of narcotic substances. We need not, therefore, be surprised that opium, administered in enormous quantities, in this painful disease, has had but little effect. I was assured by a physician, with whom I formed an acquaintance in Edinburg some years ago, and who is known, I find, to a student now present, that his own wife, while labouring under a tetanic affection, swallowed, in twenty successive days, upwards of 40,000 drops of laudanum, which is at the rate of more than two ounces a day; in all, more than an imperial quart. The lady recovered. A case is recorded in the 2d volume of the *Medico-Chirurgical Transactions*, in which an ounce of *solid* opium was taken. in divided doses, every day, for 22 days. This appears a more astounding instance than the former; but I am not sure that it was so; for, in this complaint, solid opium does not always dissolve in the stomach. I have heard the late Mr. Abernethy say that he had found enough undissolved pills of opium in the stomach after death, to poison a dozen healthy persons. This fact should teach you, if you resolve on trying opium at all, to exhibit it in a liquid form; laudanum, or a solution of the acetate, or of the muriate of morphia. And it would be well, I think, to combine the external use with the internal administration of opium; to blister the spine, and strew the blistered surface with powdered acetate of morphia, while you give it in solution by the mouth.

It is sometimes a difficult matter to introduce medicine by the mouth, so strong is the spasmodic contraction of the muscles that close the jaws. You cannot get the mouth open. Some persons set to work to heave it open, by levers; and it has been proposed, and I believe practised, to break off or extract a tooth or two, to make a passage for the introduction of medicine and of nourishment; but I hope you will never be guilty of such clumsy barbarity as this. Food, and physic, may be carried into the fauces or into the stomach by meaus of a flexible tube; and this may be inserted through the nostril; or through the mouth, by passing it between the jaws, behind the back teeth, where there is always an aperture that will admit a sufficiently large tube.

After all, in respect to the cures that have been ascribed to the opiate treatment, they have all (so far as I know) occurred in cases of milder or more chronic tetanus; and mostly in the idiopathic form of the disease; and this circumstance makes it a question whether they were *cures* at all: whether they were not simply recoveries.

Dr. William Budd (in the paper already referred to) challenges the propriety, on physiological principles, of giving any opium in this disease. He says "it has been ascertained that the effect of that drug is to excite, and not to quiet, the motor function of the spinal cord: indeed, it is well known that the motor acts of the cord may be rendered much more active and powerful, by giving, before decapitation, opium to animals that are to be subjects of experiment." He considers "these objections, furnished by theory, to be motives sufficient for the future exclusion of opium from the treatment of tetanus."

I had long been aware that the effect of opium upon frogs was to produce tetanic spasms. But in no case of poisoning by opium in the human subject (and I hava seen a great many) have I ever witnessed any approach to tetanus: and I much question the safety of arguing, in such matters, from what we know to happen in the inferior animals, to what we suppose would happen in man.

The failure, however, of opium in the severer forms of the malady, and its equivocal utility in any, taken together with these theoretical objections, prevent my recommending opium as a remedy for tetanus.

*Blood-letting.*—What is the result of experience in regard to blood-letting in tetanus? I am afraid that, as a curative agent, it has very little power over the disease. Yet it may be, and probably is, of considerable use as an auxiliary to other measures. When the disorder bears any aspect of inflammation—when, for instance, fever is lighted up, and pain is felt along the course of the spine, or when the approach of the spasm is marked by the supervention or the increase of pain in the wound—our chance of doing good by venesection is the greatest. Some of the cases that happened in the Peninsular war, were decidedly benefited by blood-letting practiced under such circumstances. I need scarcely say that though the bleeding, when adopted, should be early, free, and full, so as to produce some sensible impression upon the system, yet we must always use this remedy with caution. The tendency of the disease is to exhaust the power of the heart; and if by one over-bleeding we bring that organ to a stand-still, it may refuse to begin again.

In a complaint which depends so much on irritation, and so often on manifest irritation of extended parts, we look naturally to the *warm bath* for help. And it has been fairly tried: and some persons have found it useful; and others have found it useless, doing neither good nor harm; and some have condemned it as actually hurtful.

The *cold bath* has been extolled as a much more powerful agent than the warm; and so, doubtless, it is. But it is more potent for harm as well as for good. For example; a tetanic patient, in St. Thomas' Hospital, was plunged into a cold bath, at his own request. "All the symptoms disappeared (says Mr. Morgan) in a moment; and he was almost immediately taken out of the bath; but he was taken out lifeless." Sir James M'Grigor says that, during the campaign in Spain, "the warm bath gave only momentary relief; and the cold bath was worse than useless."

However, the application of cold water to the surface has, in many recorded instances, been of at least temporary benefit and comfort; and, in the West Indies, where the disease is common, the cold affusion still continues, I believe, to be the most favourite expedient. After it, the patient is rubbed dry, put to bed, and has laudanum administered. I have again to observe, of this remedy also, that it is chiefly serviceable in the idiopathic form of tetanus. It has been tried upon animals. Dr. Parry says that it was quite unavailing in the case of certain lambs that had the disease. In a note which I made at the time of Mr. Abernethy's lecture on Tetanus, I find the following statement. "The effect of cold in diminishing excessive muscular action was strikingly shewn in the case of a horse belonging to Professor Coleman, which had tetanus. The animal was slung, and carried out of the stable, and laid on the snow, which was then on the ground: and he was covered over wlth snow also. A horse affected with tetanus is a curious sight. His legs straddle and become stiff; his ears are pricked up; and his tail sticks out. In this case, on the application of the snow, his ears sunk, his tail became pliant, and the rigidity of his muscles was removed. He was again taken into the stable, and the spasms returned." Mr. Abernethy said, that were he himself the subject of tetanus, he would desire to have the cold affusion tried. If you are willing to assay the same remedy, do not plunge your patient into a cold bath, but take him out of his bed on an extended sheet, splash him well with cold water, wipe him dry, and place him in another dry bed. This will often, for a time at least, diminish the spasmodic action; and the patient will sometimes sleep comfortably after it.—*Ibid.*

*On the Treatment of Pseudarthrosis.* By Drs. Woppish and Bauer.—Two cases are here related for the purpose of pointing out the advantages of a long-neglected method of treating false joints, namely, the rubbing together of the ends of the bones. With the exception of the mode of treatment, the cases present nothing of particular interest. In the first, the portions of a fractured thigh which had not united in fifteen weeks were violently rubbed together for a quarter of an hour; the operation gave great pain, but it was followed by such inflammation that the bones united accurately and firmly after a few weeks more. In the second case the fracture (a compound one of the leg) had not united at the end of the eighth week; the same treatment was adopted with an equally favourable result; (but it may be a question whether patience alone might not have obtained the same end.)—*Ibid, from Med. Zeitung.*

